# Ensembles of Deep Learning Models and Transfer Learning for Ear Recognition

**DOI:** 10.3390/s19194139

**Published:** 2019-09-24

**Authors:** Hammam Alshazly, Christoph Linse, Erhardt Barth, Thomas Martinetz

**Affiliations:** 1 Institute for Neuro- and Bioinformatics, University of Lübeck, 23562 Lübeck, Germany; linse@inb.uni-luebeck.de (C.L.); barth@inb.uni-luebeck.de (E.B.); martinetz@inb.uni-luebeck.de (T.M.); 2 Mathematics Department, Faculty of Science, South Valley University, Qena 83523, Egypt

**Keywords:** ear recognition, deep features, convolutional neural networks, transfer learning, ensemble models, feature visualization

## Abstract

The recognition performance of visual recognition systems is highly dependent on extracting and representing the discriminative characteristics of image data. Convolutional neural networks (CNNs) have shown unprecedented success in a variety of visual recognition tasks due to their capability to provide in-depth representations exploiting visual image features of appearance, color, and texture. This paper presents a novel system for ear recognition based on ensembles of deep CNN-based models and more specifically the Visual Geometry Group (VGG)-like network architectures for extracting discriminative deep features from ear images. We began by training different networks of increasing depth on ear images with random weight initialization. Then, we examined pretrained models as feature extractors as well as fine-tuning them on ear images. After that, we built ensembles of the best models to further improve the recognition performance. We evaluated the proposed ensembles through identification experiments using ear images acquired under controlled and uncontrolled conditions from mathematical analysis of images (AMI), AMI cropped (AMIC) (introduced here), and West Pomeranian University of Technology (WPUT) ear datasets. The experimental results indicate that our ensembles of models yield the best performance with significant improvements over the recently published results. Moreover, we provide visual explanations of the learned features by highlighting the relevant image regions utilized by the models for making decisions or predictions.

## 1. Introduction

Automated identity recognition based on physical characteristics of humans (i.e., biometrics), such as the face, iris, fingerprints etc., has attracted a considerable amount of attention in the biometric community due to increasing security requirements. In order to be a valid biometric modality, these characteristics should be distinctive, universal, permanent, quantifiable, and convenient to measure [1]. Out of all biometric characteristics, the human face has been extensively studied. However, recognition systems based on faces have to cope with sensitivity to various image distortions including illumination changes, different poses, occlusions, aging, and facial expressions. The human ear is an emerging biometric modality which offers immense potential by being relatively invulnerable to these variations due to its stable structure.

**Figure 1:**
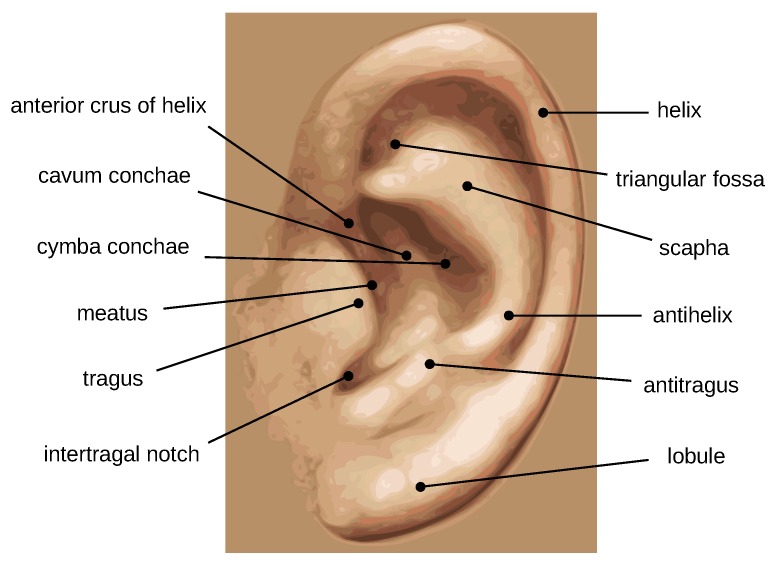
Morphological parts of the human ear.

Figure 1 illustrates the outer and inner parts of the ear structure. The outer shape contains a set of important morphological features like the tragus, anti-tragus, outer helix, anti-helix, and lobule; while the inner structure is formed by numerous valleys and ridges, all together forming a complex structure and providing discriminatory power. In addition, some other advantages of ear images include ease of acquisition, lower level of intrusiveness, and high immunity to privacy and hygienic issues. These characteristics indicate the potential of using the ear modality in personal identification.

The first large-scale study on using ear characteristics to uniquely recognize individuals was conducted by Iannarelli [2], in which he manually measured the distances between 12 discriminative biological characteristics of the ear and provided a collection of more than 10,000 ear images. The conclusion of his experiments was that no two ears were found to be identical. Since then, many researchers have been interested in exploring this emerging biometric modality and finding robust ways to represent ear images and extract their distinguishable features for constructing personal identification systems. For chronological developments of ear recognition techniques, several surveys and reviews exist, summarizing the achievements, limitations, and challenges encountered [3,4,5,6].

The vast majority of existing ear recognition systems have been constructed by employing handcrafted feature extraction methods (i.e., descriptors) to represent the ear images and a traditional classifier to match and classify the resulting feature vectors. Descriptor-based recognition techniques still offer acceptable performance for small-sized datasets acquired under controlled conditions although these descriptors can only handle specific image variations. In earlier studies, multiple ear image characteristics including texture, edges, shape contours, and gradient information were utilized to better describe the ear features. For instance, texture descriptors have been extensively studied in ear recognition, justifying the importance of texture cues when featuring ear images [7,8,9]. On the other hand, methods exploiting gradient magnitude and orientation are also considered for a better description of the ear shape and contour [10,11,12]. The color information is also explored for ear recognition as discussed in [13] and by fusing features extracted at different color spaces in [14]. However, these methods are still limited in their ability to leverage multiple characteristics of ear images. More important is the performance degradation of these techniques when the images are taken under uncontrolled conditions with a wide range of pose and illumination variations as well as occlusions which are common in real-world applications [15].

Nowadays, deep learning [16], a type of machine learning techniques in which the models learn discriminative features at multiple levels of abstractions and perform recognition tasks directly from raw data, has influenced the field of computer vision significantly. At the core of deep learning techniques are convolutional neural networks (CNNs) which have achieved astonishing breakthroughs in a variety of visual recognition tasks including image classification [17,18,19], object detection [20,21,22], face recognition [23,24,25,26], and the like. The key factors behind the success of these methods are the large-scale and publicly available image databases such as ImageNet [27] and the advances in high-performance hardware devices such as GPUs for training such models in a reasonable time. For some application domains, such as image classification and face recognition where decent amount of data is available, CNN-based systems have advanced the state-of-the-art performance to extraordinary levels. However, in other applications or domains where data is limited or expensive to collect, the benefits of deep models and powerful CNN-based representations are not fully utilized. Among these domains is ear recognition, which has potential usage in forensics and security applications. As a consequence, ear recognition technology is lacking behind other biometric-based technologies such as face recognition due to the imposed restrictions of insufficient large-scale labeled ear databases.

A first step towards building CNN-based ear recognition models is introduced in [28]. The authors considered three CNN architectures, AlexNet [17], VGG-16 [18], and SqueezeNet [29], and explored different training strategies using extensive techniques of data augmentation to overcome the lack of abundant training data. Two learning strategies were applied: full model learning and selective model learning. In the first approach, the models are trained from scratch with increasing levels of data augmentation; while in the second approach, the models are initialized from pretrained models learned on the ImageNet dataset and then are fine-tuned using the training part of the ear dataset. The best results came from the model based on fine-tuning a pretrained SqueezeNet model which achieved a recognition rate of 62%. Ying et al. [30] proposed a deep CNN model for ear recognition considering various aspects, such as network size, regularization methods, and activation functions, in order to improve the model performance. The model was tested under occlusion and rotation and showed a satisfactory recognition rate when the degree of distortions was small. The authors in [6] proposed a standard CNN-based ear recognition system and several models were trained using ear images acquired under controlled conditions of lighting, quality, size, and viewing angles. Their system obtained good results when recognizing ear images similar to what the model was trained on. The authors in [31] proposed an ear recognition system by designing a deep convolutional network that has three convolutional layers each followed by a max pooling layer and two fully connected layers. To test their model, they used the University of Science and Technology Beijing III (USTB III) ear dataset [32] which has 79 subjects. Even though their model showed a good performance using a small-sized dataset, they emphasized that ear recognition under unconstrained conditions such as pose variations and occlusion is a challenging task especially when there is insufficient ear data.

An effective solution to address ear recognition when training data is limited is to use transfer learning [33,34,35]. For instance, an ear recognition system was constructed based on transfer learning from pretrained AlexNet [36]. The training and testing of their model were performed on ear images acquired under controlled lighting conditions by a smart-phone for 10 subjects and only 300 images. Transfer learning is also extensively explored in addressing ear recognition challenges under unconstrained imaging conditions in [37,38,39,40]. The authors in [41] investigated the impact of fusing manually extracted features based on some well-known descriptors and CNN-based learned features for improving ear recognition. Their experimental results suggested that handcrafted and learned features are complementary as their combination resulted in the best performance [41]. A comparative analysis of several handcrafted features and three deep learning-based methods is presented in [42]. The authors carried out identification experiments on the Annotated Web Ears (AWE) database [5] to assess the sensitivity of both models to specific covariates. The obtained results indicated the negative impact of severe head rotation and occlusions on the recognition performance compared to other covariates such as gender and ethnicity.

In 2017, Emeršič et al. [43] announced the first unconstrained ear recognition challenge (UERC), an effort to evaluate existing ear recognition techniques on an extended version of the AWE ear dataset (also named UERC) gathered specifically for the challenge. The UERC dataset contains 11,804 ear images for 3706 subjects and all the images were collected from the web under unconstrained conditions. In total, six techniques were submitted and two models were given by the organizers. Four recognition methods utilized VGG-based models but with different preprocessing pipelines (i.e., flipping and alignment) and various ways of featuring (i.e., activations from a selected output layer). They presented a comparative analysis of different covariates, such as occlusion, rotation, spatial resolution, and gallery size, on the recognition performance, and the obtained results highlighted insightful findings summarized in [43]. The second round of the challenge, UERC 2019 [44], evaluated 13 ear recognition techniques. The majority of the submitted approaches utilized deep learning techniques. More interesting, around 50% of the approaches were based on multiple ear image representations due to limitations of a single model representation to capture the wide and complex appearance variability. Even though the obtained results show some improvements in the recognition rate when using ensembles or multiple descriptor combinations, the problem of recognizing humans from ear images under unconstrained conditions has not been solved and more research is needed [44]. The winner of the challenge was ScoreNet-5 [45] which utilizes a fusion learning approach.

Despite the rapid increase and wide adoption of deep learning models in various domains, their internal functionality is not fully understood and hard to interpret because of the black-box nature of deep networks [46]. Thus, to understand what deep networks have learned is of a great importance. By doing so, the obtained results become more interpretable and also provide insights into their success. Recently, some studies have been focused on visualizing and interpreting the learned features from CNN-based models in order to build trust and reliability in their predictions as well as to obtain insights to better design network architectures [47,48,49]. The first work on feature visualization was introduced by Zeiler et al. [47] to understand the functionality of internal layers and what types of features they learn. However, the visualization process is performed by employing a deconvolutional network (DeconvNet) to project the activations back to the input pixel space and highlight the certain patterns responsible for a given activation of interest in the feature maps. The visualization is provided only for convolutional layers and ignores the fully connected ones. In [50], a generalization to the DeconvNet approach is proposed which can be applied to visualize the activations of any layer rather than only the convolutional ones. Zhou et al. [49] proposed another visualization approach called class activation map (CAM) to produce a localization map for CNN-based models. CAM is mainly used to identify the important image regions by projecting back the weights of the last layer onto the convolutional feature maps. However, it cannot be used for networks with multiple fully connected layers before the last layer. Gradient-weighted class activation mapping (Grad-CAM) [51] generalizes the CAM approach to visualize any CNN-based network architecture. Grad-CAM provides visual explanations to understand CNN-based models and makes them more transparent by highlighting the important regions in the input image leading to certain predictions. In our experiments, we considered the Grad-CAM approach for visualizing the learned features by the visual geometry group (VGG)-based models and provide comprehensive analysis of the obtained results.

In this paper, we choose the VGG network as one of the top performers in both image classification and object localization challenges [27], and introduce configurations to suit ear recognition on the used datasets. We experimentally investigate selecting the optimal input image size and explore different regularization techniques to help the models generalize and alleviate overfitting. Three learning strategies, namely training from scratch with randomly initialized network weights and two transfer learning strategies from pretrained VGG-based models, are investigated. When training our models, we employed a set of different augmentation techniques to increase the number of training samples. The best obtained models from these learning strategies were then utilized to build ensembles of models with varying depth to improve performance. Moreover, we provide visual explanations of the learned features by the models with the Grad-CAM visualization technique to better interpret the obtained results. Overall, this paper exhibits the following contributions:

- We propose ensembles of VGG-based models that outperform other ear recognition techniques on the considered mathematical analysis of images (AMI) and West Pomeranian University of Technology (WPUT) ear datasets. Extensive experiments using different learning methodologies were carried out and a comprehensive analysis of their performance and computational complexity is presented;
- We present a novel ear image set, known as AMI cropped (AMIC), based on the original AMI dataset with substantial background and profile regions removed; this is intended to be used to evaluate the performance of different ear identification and verification systems under challenging conditions;
- We provide visualizations of the learned features to interpret the obtained results and increase the confidence and reliability in the models’ predictions, which is useful when working with a limited amount of training data.

The remainder of the paper is structured as follows. Section 2 describes the VGG network architectures and presents our configurations to suit the target problem. In Section 3, we discuss the learning strategies followed to find better deep ear image representations. The experimental setup is explained in Section 4. The obtained results along with the Grad-CAM visualization are discussed in Section 5. Finally, Section 6 concludes the paper and gives insights into future research directions.

## 2. VGG-Based Network Architectures

The VGG network [18] is a very successful CNN architecture in image recognition and object localization challenges [52]. The architecture is inspired by conventional CNN architectures, such as LeNet [53] and AlexNet [17], but has a substantially increased depth for to improve performance. The VGG networks are designed by stacking five convolutional (conv) blocks interspersed by max-pooling layers and a fully connected block with three layers. However, it introduced some key design improvements to CNN architectures through the use of small 3×3 convolution kernels and an increase in the network depth through adding more conv layers. The additional conv layers are essential for covering an effective receptive field of the input image that leads to more discriminative features. Furthermore, using a small filter size leads to a significant reduction in the number of parameters per layer, especially in the first conv layers. Consequently, deep CNN architectures became less prone to overfitting and much more feasible to train. These significant improvements introduced in the VGG network architecture produced models that won first and second places in object localization and ImageNet classification challenges, respectively.

The original VGG network [18] uses RGB images with a fixed size of 224×224 pixels as an input size. The pixels are preprocessed by subtracting the mean RGB value computed over the training images. Each image undergoes a series of conv layers. To preserve the spatial resolution after convolution, a fixed stride of one pixel is used along with zero padding of one pixel. The max-pooling layers which follow each conv block are used to reduce the feature maps after convolution and make the resulting representations invariant to distortions and small translations. The max-pooling operations are performed over a 2×2 window size with a stride of two. The stack of conv layers is followed by three fully connected layers with 4096 neurons in the first two layers and 1000 neurons in the last layer to match the 1000 classes of ImageNet dataset. Finally, a softmax layer is used. The hidden layers are supplied with a Rectified Linear Unit (ReLU) [54] activation function to increase the non-linearity of the network architecture.

In this paper, we introduce some changes to the VGG architecture and adapt the network to better suit the ear recognition problem on the considered ear datasets. We explore four VGG-based models of increasing network depth starting from 11 up to 19 layers, although they have a design similarity for fair evaluation. We use the terminology VGG-<*i*> to refer to the different models, where *i* refers to the total number of weighted layers in the network. Table 1 illustrates the detailed configurations of the considered VGG-based models with respect to number of conv blocks, number of conv layers per block, number of filters for each layer, position of pooling layers, output size of the feature maps after each operation, and number of neurons in each fully connected layer. As a result of the variable spatial resolution of images from each dataset, we place the images into a canvas, keep their aspect ratios and scale the input images accordingly. We found that the optimal canvas sizes are 133×190 for both the AMI and AMIC datasets and 170×220 for the WPUT dataset. We use convolution kernels of size 3×3 with a stride of one for all conv layers. Our max-pooling operations have a kernel size of 2×2 and a stride of two. The last max-pooling operation before the first fully connected layer is replaced by adaptive average pooling, which reduces the feature map resolution to 5×5 and makes the model applicable to various input sizes. Subsequently, we flatten the feature maps and use their activations as input for the first fully connected layer. We regularize our models with 50% dropout [55] between the three fully connected layers. To account for the effect of having only 100 and 474 classes instead of 1000 in the datasets, we shrink the first two fully connected layers to 2048 dimensions and adapt the size of the last layer accordingly.

## 3. Learning Ear Image Representations

The performance of recognition systems is highly dependent on the robustness and discriminative ability to represent image information. Over recent years, deep CNNs have shown a powerful capability to learn discriminative representations from images that summarize their essential factors of variations. However, learning these rich representations by deep CNN models requires a massive amount of labeled training data and powerful computational resources to train these models and optimize the large space of hyperparameters. If these requirements represent an issue for any specific application or domain, learning these representations from scratch would be unfeasible [56]. One effective solution for these limitations is to transfer the learned representations by deep models from similar visual recognition tasks building on the hypothesis that CNN-based features are transferable [21,34,35,57,58]. Nevertheless, the ideal method of feature transfer has not yet been found because the high-level layers of most deep CNN-based models tend to learn data and task specific features. This paper considers CNN-based representations and specifically VGG-based networks of different depth to construct ear recognition systems. To this end, we explore three representation learning methodologies discussed in the following subsections and select the best representation method to build ensemble models to further improve the recognition performance.

### 3.1. Training with Random Weight Initialization

One particular advantage of deep learning methods is learning hierarchical data representations to form certain levels of abstractions. In the scope of image classification problems, lower layers process rather basic relations between pixels and basic textures, while deeper layers consider high-level information including semantic relationships. Despite the advantages and performance gain obtained by deeper network designs, increasing the network depth increases the number of trainable parameters that need to be optimized during training. The latter problem becomes more obvious in the case of dealing with smaller datasets, and more specifically, when these parameters are randomly initialized. In this paper, we use ‘training with random weight initialization’ and ‘training from scratch’ interchangeably. In order to enhance the generalization ability of the models and alleviate the overfitting problem, we adopt several regularization strategies, including dropout [55] and data augmentation. These regularization techniques help the models to generalize well with unseen data and improve the overall recognition performance.

### 3.2. Pretrained Models as Feature Extractors

Another effective strategy of using deep models to overcome the limitation of small-sized datasets is initializing the network with weights from a pretrained model instead of random weight initialization. By doing so, we benefit from the robust and discriminative filters learned by top-performing CNNs on large-scale datasets such as the ImageNet dataset [27]. These pretrained models can be used to perform feature extraction tasks considering that CNNs are composed of a convolutional feature extraction part and a fully connected network for classification. Consequently, these models can be utilized as generic feature extractors [59,60]. Similarly, the VGG networks consist of a feature extracting convolutional part followed by a classification block with three fully connected layers. The first layers of the convolutional part tend to extract low-level features such as edges, color, and texture, whereas the high-level layers tend to capture more abstract high-level features. Transferring the learned features across different recognition tasks is achieved by freezing specific parts of the learned models and feeding the extracted features into a fully connected network or traditional classifiers [21,34,35,57]. In this work, we follow a similar approach to investigate whether the learned ImageNet features actually suit the ear recognition task. We freeze the weights of all convolutional layers and retrain only the classifier on top (i.e., the fully connected layers) using the training part of each ear dataset.

### 3.3. Fine-Tuning Pretrained Models

Using the fixed representations learned on ImageNet dataset for general object recognition might not be the optimal choice for the specific task of ear recognition. Hence, in our third learning strategy, we adjust all layers, including the convolutional ones and further train the models using the training set of each dataset. This fine-tuning or adaptation of pretrained models helps them to see and understand the ear’s shape and structure, thus leading to better and more discriminative features and increased overall recognition performance. Indeed, it is one of the most widely used approaches of transfer learning from CNN-based models and has the advantage of accelerating the training process.

### 3.4. Ensembles of Deep Models

**Figure 2:**
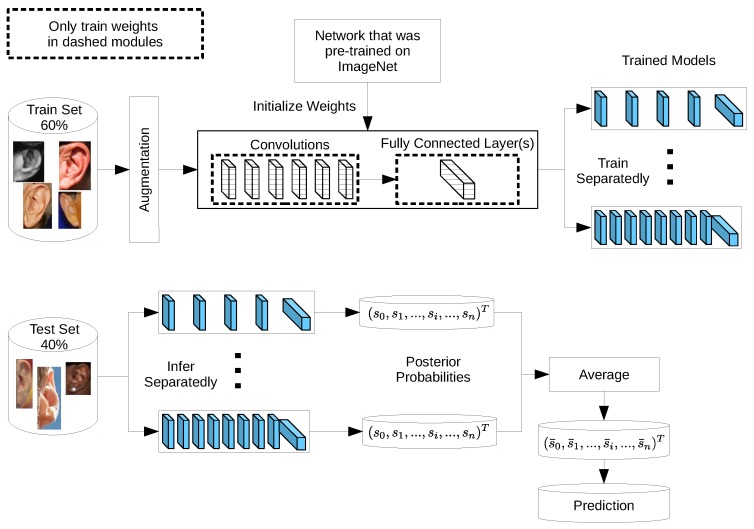
A schematic diagram showing the process of constructing an ensemble of deep models for improving ear recognition. The models are trained separately and the best performing ones are chosen for the different combinations.

Ensemble models are powerful machine learning techniques which improve the overall performance by combining the decisions from multiple models. As a result of the stochastic nature of deep neural networks where each model learns specific filters and patterns, building ensembles of deep models is an effective solution to boost the recognition accuracy. Ensembles have been adopted to compete in many recognition challenges [17,18,44,61,62]. Herein, we propose ensembles of deep models based on VGG-like network architectures of various network depths to improve ear recognition accuracy. Figure 2 displays a complete overview of the ensemble construction process. There are two essential stages. First, we use 60% of each dataset for training or fine-tuning the different networks separately to obtain the trained models. Second, we select the best performing models and construct ensembles of these models to classify the remaining 40% of the dataset. On the basis of our experimental results, which indicate the superiority of fine-tuned models over other learning methods investigated in this work, fine-tuned models are considered for building ensembles through the formation of voting committees. Each test image is passed through the considered models and each model provides a class membership score in terms of posterior probabilities. The individual votes are combined by averaging the posterior probabilities and the final prediction is considered as the class with the highest probability in the averaged vector (see Figure 2). While building ensembles is an effective way for boosting the overall prediction accuracy, it comes at a computational cost, as instead of training a single network, we must train multiple networks.

## 4. Experimental Setup

In order to analyze the performance characteristics of the suggested VGG-based network architectures in ear recognition, we conducted a series of identification experiments on three benchmark datasets. Four VGG-like network variants with increasing depth (11, 13, 16, and 19 layers) were implemented and compared to each other using three different learning strategies. Thereafter, we selected the best performing models to build ensembles to boost the recognition performance.

For the AMI and AMIC datasets, the learning rate was scheduled to have an initial value of 0.01 and to decrease depending on the learning strategy. In the case of training the networks with random weight initialization, the learning rate was divided by 2 every 100 training epochs. In the other two strategies, the learning rate was halved every 50 epochs due to their fast convergence. For the WPUT dataset, the learning rate was set initially to 0.02 and was divided by 5 every 300 epochs when training the networks from scratch and divided by 5 every 100 epochs for the other two strategies. To improve the models convergence during training, we applied batch normalization after each convolution layer. The networks were trained using back-propagation [63] and optimized by applying stochastic gradient descent with a momentum of 0.9 on a cross-entropy loss.

The models were trained on a PC with Intel(R) Core(TM) i7-3770 CPU, 8 MB RAM and Nvidia GTX 1080 for 500 epochs (random weight initialization), 200 epochs (training only fully connected layers), and 150 epochs (fine-tuning all weights) until convergence. However, the number of training epochs for the WPUT dataset was 900 for scratch training and 300 for the other two strategies.

The following subsections give more details about the ear image datasets used and the data augmentation methods followed in this work to improve recognition accuracy and avoid overfitting of the training data by the deeper models. Furthermore, the experimental protocol and performance metrics and curves used to evaluate the models’ performance are explained.

### 4.1. Ear Datasets

**Figure 3:**
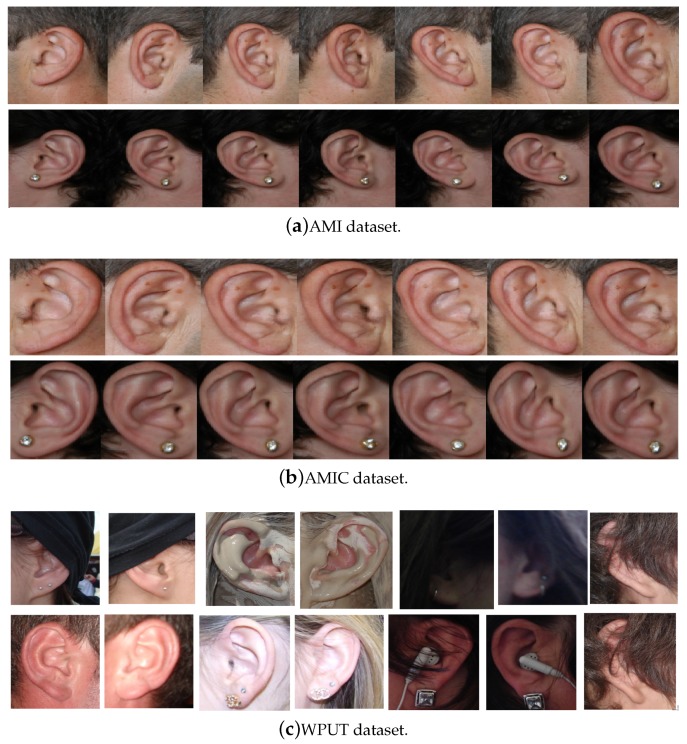
Sample images for two distinct subjects from the AMI and AMIC ear datasets, where images in the bottom (**b**) are tightly cropped from the profile images shown at the top (**a**); while images in (**c**) show ear images of selected individuals to illustrate the wide variability of image distortions that exist in the WPUT ear dataset.

The AMI ear database [64] was collected from 100 students and teaching staff from the Computer Science department at University of Las Palmas de Gran Canaria (ULPGC), Las Palmas, Spain. It contains ear images acquired from both males and females of different ages, ranging from 19 to 65 years. In total, the database contains 700 images, whereby each subject has exactly seven images. Out of the seven images, six show the right ear and one image shows the left ear. For each subject, the right ear is captured with the head facing forward, looking up and down, and looking left and right. The sixth image of the right profile is taken with the subject facing forward with a different focal length. The last image is a left side profile where the subject is facing forward. All images were acquired in an indoor environment and under the same lighting conditions. The images have a spatial resolution of 492 × 702 pixels and are available in JPEG format. Sample images from the AMI database are illustrated in Figure 3a. Although the AMI dataset contains good quality ear images and exhibits different pose variations, other parts of the profile images such as the hair, and parts of neck and face are visible. In order to alleviate the influence of these parts on the ear recognition performance, we tightly cropped all profile images from the AMI dataset and prepared a new image set named AMIC where only the ear structure is visible, as can be seen from Figure 3b. Because the images in the original AMI dataset are taken with different head poses, the cropped images have various spatial resolutions. In order to introduce more variability to the AMIC dataset, the images are not aligned.

The third dataset considered in our study is the WPUT ear database [65]. Its images were acquired from both genders for the right and left ears. The available dataset has 3348 ear images for 474 subjects with 1388 duplicated images. For our experiments, we used a cleaned version of the dataset which has in total 1960 images for 474 subjects, each individual having between four to eight ear images. All images have a spatial resolution of 380×500 pixels and exhibit a wide range of image deformations. The images were taken under different lighting conditions and various viewing angles. Occlusions by hair, headdresses, ear-pads, earrings, and other accessories represent real challenges related to the WPUT dataset. These significant deformations make the WPUT database prominent to evaluate biometric recognition models under unconstrained conditions. Figure 3c shows sample images from the WPUT ear dataset.

### 4.2. Data Augmentation

Since the process of training deep CNNs requires a huge amount of labeled training samples to reduce overfitting, and given the fact that existing ear datasets are still limited (i.e., a few hundred to a few thousand images), profound data augmentation is a crucial measure to effectively increase the number of training samples. Appearance variations are artificially introduced without any extra labeling costs by using label-preserving transformations [66,67,68]. We combined various forms of data augmentation into a single preprocessing pipeline to transform the original images to generate variants. The augmented images were generated on the spot before being fed into the models. In this work, we optimized our dataset augmentation to improve the accuracy of the VGG network-based models; the following is a list of image transformations applied on each training image in the ear datasets:

- Random scaling to fill 70–100% of the canvas area;
- Random rotation from −45 to +45 degrees;
- Random horizontal shearing in the range of −5% to 5% of the image width;
- Random cropping, removing up to 10% of the image, keeping the aspect ratio;
- Resizing to fit the canvas;
- Gaussian blur with radius of 3 and 50% chance;
- Blending Gaussian noise of random amount;
- Random change of brightness from −20% to +20%;
- Random change of contrast from −40% to +40%;
- Random change of saturation from −20% to +20%;
- Random change of hue from −5% to +5% of the color range;
- Horizontal flipping with 50% chance;
- Normalization by subtracting the mean pixel and division by its standard deviation.

### 4.3. Evaluation Metrics and Protocols

In order to evaluate performance for all identification experiments, we plotted and computed the following performance curves and metrics: (i) Cumulative match characteristic (CMC) curves for each identification experiment to visualize the fine performance differences between the different models; (ii) rank-one (R1) and rank-five (R5) recognition rates; and (iii) area under the CMC curve (AUC).

We followed the same experimental protocol for splitting each dataset into two disjointed sets: a training set, and a test set covering 60% and 40%, respectively. We trained or fine-tuned the models on the training set and evaluated their performance on the test set.

## 5. Results and Analysis

**Figure 4:**
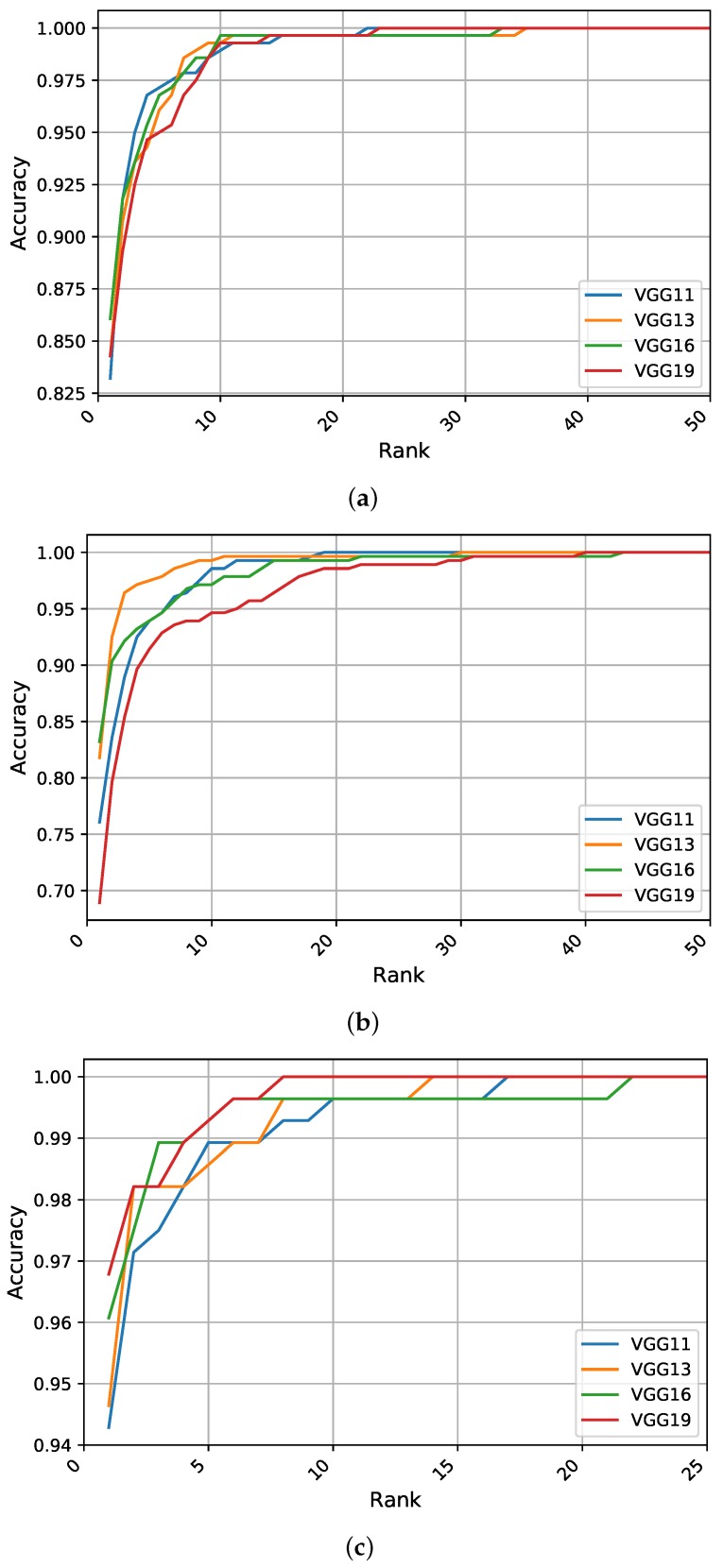
The cumulative match characteristic (CMC) curves generated in the identification experiments summarizing the recognition performance of the different models under (**a**) scratch training, (**b**) feature extraction, and (**c**) fine-tuning on the test set of the AMI ear dataset.

**Figure 5:**
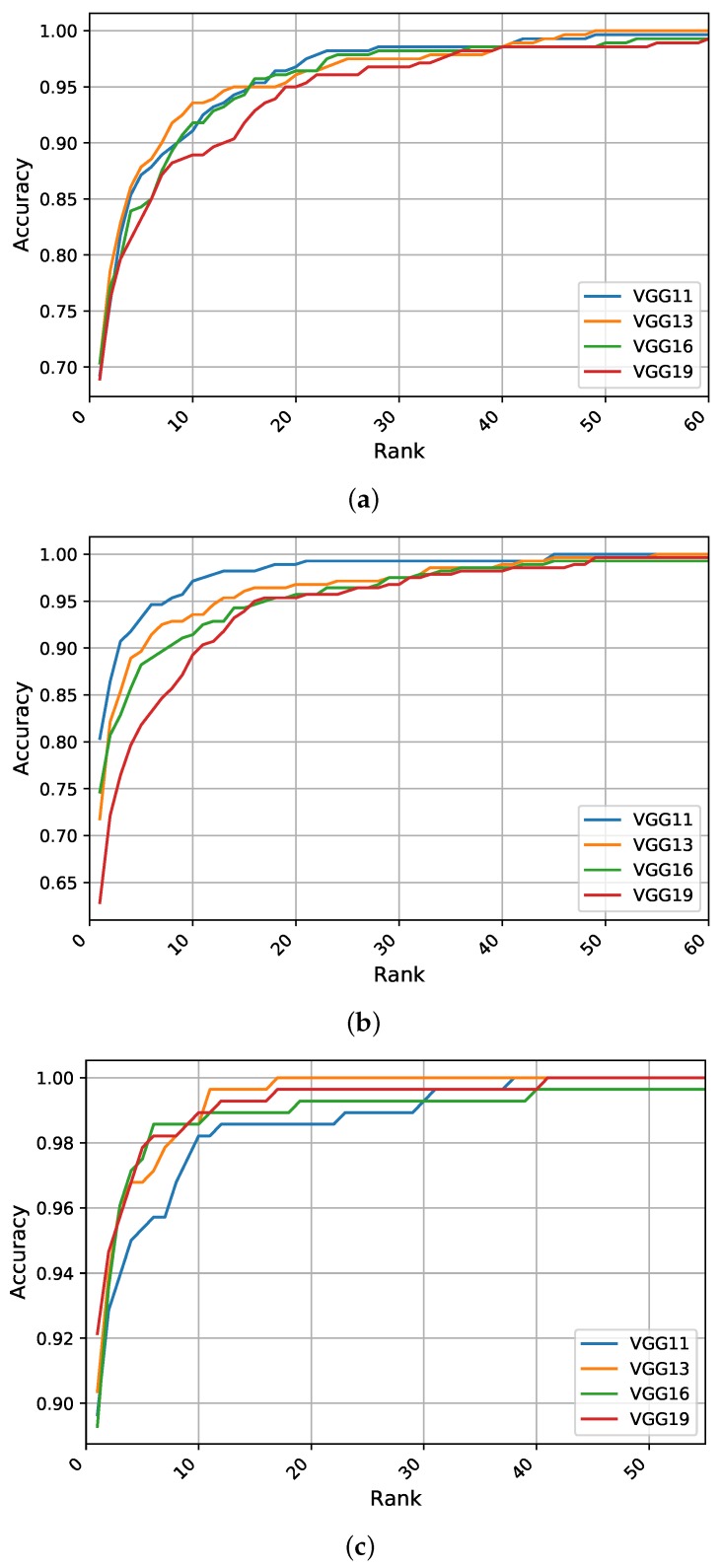
The CMC curves generated in the identification experiments summarizing the recognition performance of the different models under (**a**) scratch training, (**b**) feature extraction, and (**c**) fine-tuning on the test set of the AMIC ear dataset.

**Figure 6:**
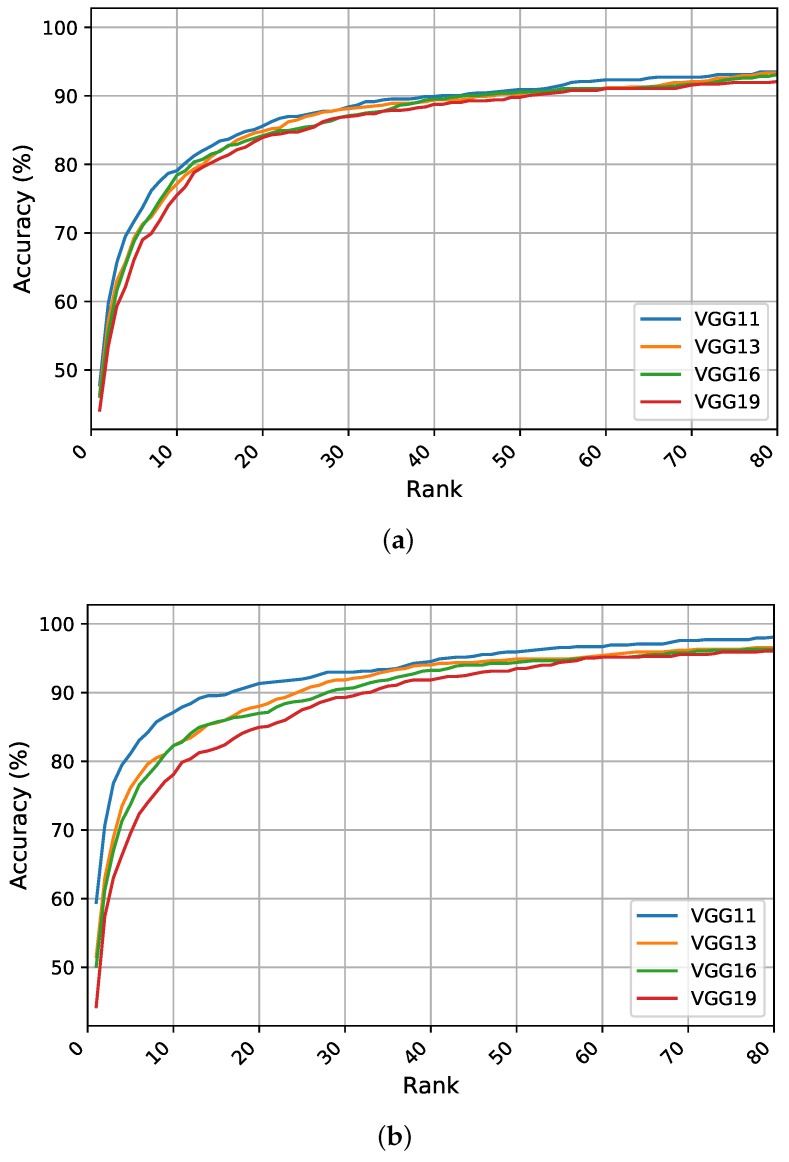

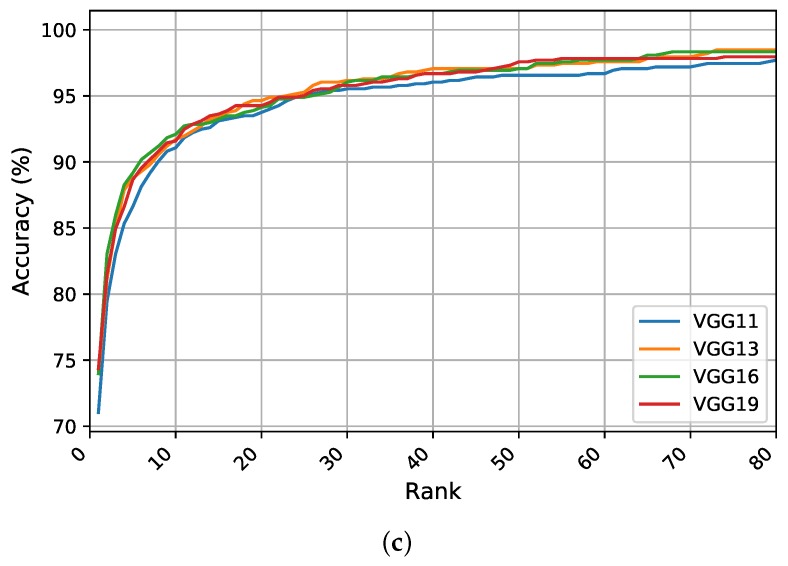
The CMC curves generated in the identification experiments summarizing the recognition performance of the different models under (**a**) scratch training, (**b**) feature extraction, and (**c**) fine-tuning on the test set of the WPUT ear dataset.

This section reports the results of our identification experiments using the different models and strategies on the aforementioned ear datasets. Table 2 summarizes the obtained results using the three quantitative metrics of R1, R5, and AUC, where the highest performance metrics for each strategy on each dataset are shown in bold. We also report the values of the evaluation metrics when building ensembles of models and compare our results with state-of-the-art methods in the literature, as given in Table 2. Additionally, to visualize the performance differences of the different models, the CMC curves generated for each identification experiment are presented in Figure 4, Figure 5 and Figure 6.

In the following subsections, we first present the obtained results under the different learning strategies using the original AMI dataset. Then, we discuss the obtained results on the AMIC dataset and assess the effect of cropping auxiliary parts from the profile images on the recognition performance. Thereafter, we evaluate the models in real-world scenarios associated with ear recognition (i.e., in unconstrained conditions or in the wild) using the WPUT dataset. On the basis of the obtained results, we select the best performing models and explore various model combinations to build ensembles and analyze the impact on the performance metrics. Finally, with the help of Grad-CAM visualization, we present visualizations of the discriminative image regions learned by the models for inferring predictions.

### 5.1. Identification Experiments on the AMI Dataset

Our objective in this section is to measure the influence of increasing the network depth on the recognition performance when using the AMI dataset in a closed-set identification scenario. Here, we discuss the experimental results attained with our three feature learning strategies.

**Scratch Training:** The identification experiments when training a network with randomly initialized weights suggest that the VGG-16 is the top performer with R1 of 86%, while the VGG-13 and VGG-19 models perform in a similar manner. However, for the other performance metrics of R5 and AUC, the leader is the shallower model VGG-11, indicating its competitive performance in the higher ranks. Figure 4a illustrates the CMC curves for the obtained models. We can see that VGG-16 has the highest performance although the difference in performance between the models is relatively small. In addition, the VGG-16 model is able to identify above 99% of the test images within the top 10 ranks.

**Feature Extraction:** When freezing the weights of all convolutional layers and tuning only the fully connected layers of the pretrained models, we observe a worsening in the learning process compared to the above-mentioned strategy. Here, the VGG-16 network performs the best with respect to R1, exceeding 83%, while the VGG-13 model becomes more competitive and outperforms all other models with respect to the other two metrics (R5 and AUC) due to its enhanced performance in the higher ranks. Figure 4b shows the CMC curves when utilizing the pretrained models as feature extractors, we observe a noticeable difference in the recognition curves compared to the previous strategy. Surprisingly, the competitive performance of the VGG-13 in the higher ranks increases from 81.78% for R1 to above 96% for R2, continuing to lead the performance within the top 20 ranks. The CMC curves in Figure 4b indicate the superiority of the shallower models over deeper ones when using small-sized datasets and when using these methods of transfer learning from the CNN-based models.

**Fine-Tuning Pretrained Models:** Giving all layers of the pretrained models a chance to optimize their weights using the training part of the AMI dataset, we observe a noticeable improvement in recognition performance and convergence time for all models. In particular, the deeper models exhibit improvements with a large jump of above 10% in R1 recognition rates. This consistent phenomenon only holds if both the weights of the fully connected layers and the convolutional feature extraction part are adjusted to the problem, making the best performing model (i.e., VGG-19) achieve R1 recognition rate of nearly 97%. In almost all cases, the performance is the highest for this learning strategy with respect to the other two metrics of R5 and AUC. When considering the CMC curves presented in Figure 4c, we notice that the performance curves for the fine-tuned models are much better compared to the other two strategies regarding the starting point of the accuracy curves and in terms of how many ranks from identifying 100% of the test images.

Overall, the obtained models achieve a similar performance in terms of R1 when trained from scratch, with a noticeable advantage over when the training was only for the fully connected layers of the pretrained models. It is also observed that when learning only specific parts of the pretrained models, the deeper networks do not improve the recognition performance and the best model under this learning strategy, the VGG-16 network, achieves 83% for R1, which is considered as good as the worst model of the first learning strategy, the VGG-11 model. Intuitively, when the network depth increases, the models can learn more complex features incorporating more semantic information. As a result, the recognition rate should increase as soon as overfitting is taken care of. However, the obtained results indicate an exception for the VGG-19 model and make us believe that VGG-19 features from ImageNet do not suit the ear recognition task well. This is noticeable when using the ImageNet features and training a stack of fully connected layers upon the convolutional layers, the performance decreases for the VGG-19 architecture, suggesting that the convolutional layers of the deeper VGG-19 architecture already show high-level characteristics which are not generic for ear recognition purposes. Nevertheless, when we include the convolutional layers in the training process we observe a great improvement in nearly all performance metrics. In particular, the deeper models benefit from the pretrained model weights to alleviate overfitting, while being allowed to adjust deep convolutional layers and to form less ImageNet specific filters.

**Ensembles of Models:** On the basis of the experimental results which indicate that models obtained using the fine-tuning strategy attain the highest recognition performance, we choose these models for constructing different ensembles. We have experimentally examined several combinations and checked their impact on the performance metrics. Table 2 presents the best three ensembles of models and their obtained results in terms of the R1, R5, and AUC metrics. We start by adding the VGG-16 to the VGG-13 model and observed an improvement of above 2% in R1. Similarly, adding the deeper model VGG-19 to both VGG-13 and VGG-16 yields the best overall performance with a result of 97.5% for R1. However, adding the shallower model VGG-11 to this triplet did not increase the recognition rate but rather cancelled the advantage of adding VGG-19 to the ensemble.

### 5.2. Identification Experiments on the AMIC Dataset

The main objective of the experiments conducted in this section was to ascertain the impact of removing auxiliary information from the profile images on training and tuning the considered deep models. The presented results in Table 2 and Figure 5 indicate lower performance metrics from the identification experiments compared with results obtained on the AMI dataset.

**Scratch Training:** When using the tightly cropped images from the AMIC dataset, training with randomly initialized weights suffers from a noticeable drop in the recognition rates compared to the AMI dataset with a margin of more than 10%. Just like before with AMI, the different models perform approximately on the same level with a minimal performance difference. The VGG-13 model achieves the highest recognition rates of above 70% for R1. Figure 5a visualizes a complete view of the recognition rates achieved by the models and emphasizes the negative effect of removing other profile parts from the images. Even though the difference between models is constrained within 1%, the CMC curves indicate an improved performance for the shallower models at higher ranks compared with the deeper models.

**Feature Extraction:** On the AMIC dataset, the VGG-11 network achieves an R1 recognition rate of above 80%, outperforming all other models with respect to the three performance metrics. The CMC curves in Figure 5b indicate the superior performance of the shallower models over the deeper ones. This emphasizes the importance of adjusting the weights from the convolutional part as retraining only a few fully connected layers is not sufficient.

**Fine-Tuning Pretrained Models:** Compared to the above-mentioned strategies, tuning all parameters of a pretrained network yields a much better performance and avoids the overfitting problem reasonably well. Even though all models are influenced by the cropping process, the improvement in recognition rates is with the large margin of nearly 22%. Herein, the VGG-19 model leads the performance in all metrics with a rate of 92.14% for R1, while the other models have similar performance for R1 of nearly 90%. Fine-tuning the convolutional block in addition to the classification block of the pretrained models leads to a superior performance. The CMC curves in Figure 5c, show an improvement in all recognition curves with higher rates for the VGG-19 in the first five ranks, then an improved performance for the VGG-16 followed by VGG-13 in the higher ranks.

**Ensembles of Models:** We also consider different combinations of models obtained on the AMIC dataset for making ensembles and measure the gain in performance metrics. We start by combining two fine-tuned models of VGG-13 and VGG-16, and we observe a slight improvement over using any of the other models separately. Surprisingly, when adding VGG-19 to the ensemble, we notice a slight improvement in the R5 and AUC metrics while getting an identical R1 recognition rate, which means that the VGG-19 model helps the ensemble to improve recognition in the higher ranks. On the other hand, adding the shallower model VGG-11 to the triplet leads to a performance gain of above 1% with an R1 recognition rate of 93.21%, which is the highest performance achieved on the AMIC dataset.

In general, learning deep representations of tightly cropped ear images seems to be more challenging, especially when the cropped images introduce variable sizes and pose variations. One logical reason for the performance drop, which will be explained in more details with the help of visualizations in Section 5.4, is that the profile images of the AMI dataset contain additional textural information, such as hair color, haircut, skin from the neck or cheek, and other visible parts, that the models are allowed to utilize to infer a prediction. Thus, removing these extra parts from the profile images yields information loss and performance degradation. In order to check whether the consistent drop originates from the AMI and AMIC image sets using the same hyperparameters for training, we did an extensive hyperparameter search for the AMIC. However, this did not lead to any significant change in the performance metrics, substantiating our hypothesis.

We also examined the model performance with respect to the network depth. For the AMIC dataset, we found that shallower models perform as well as the deeper ones as they are less prone to overfitting and have fewer parameters to optimize. Another possible interpretation for this behavior can be attributed to two things: First, on the AMI dataset, the additional information related to hair, skin, and other details increase the variance of the data and offers an incentive for learning higher level filters, which have to distinguish between haircuts, skin textures, and other details. The additional variance requires a deep network structure that is capable of learning more nonlinear and more complex filters; second, a smaller parameter space seems to be enough for this low-variance image set and should be chosen in accordance to Occam’s razor [72].

### 5.3. Identification Experiments on the WPUT Dataset

This section reports the recognition results obtained using ear images acquired under difficult or unconstrained imaging conditions. The experiments were conducted under a similar experimental protocol. Table 2 presents the results for the quantitative metrics; the CMC curves summarizing the overall recognition performance of the different models are shown in Figure 6. We start by briefly discussing the obtained results under each learning strategy.

**Scratch Training:** When training deep models from scratch on the unconstrained WPUT dataset, a significant drop in recognition rates is observed with respect to the other datasets. A logical reason for this is the lack of sufficient training data and the difficulty introduced by the wide range of image variations. Furthermore, the WPUT dataset has approximately an equal number of images for the left and right ears, each with different viewing angles which could impel the models to learn these variations and not focus on the important features. This problem emphasizes the importance of having abundant training samples for each individual to help the models learn and generalize to the level of intraclass variations. A general observation from Table 2 is that shallower models tend to learn more robust features than deeper ones due to the lower number of learnable parameters. Here, the VGG-11 model obtains the highest values for all metrics while the other models have a similar performance. Figure 6a presents the CMC curves which show the improved accuracy for VGG-11 followed by VGG-13 across all ranks. The results indicate that learning ear image features from scratch under unconstrained scenarios is difficult and using more training data is critical to learning useful features.

**Feature Extraction:** The performance of feature extraction through the pretrained models under uncontrolled conditions is better than training from scratch. One reason for this is the benefit from the learned filters in the low-level layers which extract more generic features. Additionally, the limited training images are used to tune only the classification block on top. Again, VGG-11 is the top performer with a R1 recognition rate of nearly 60%, whereas VGG-19 has the worst performance between the tested models. Therefore, under this learning representation strategy, shallower models show a clear advantage over deeper ones due to the lower number of parameters to optimize. As reported in Table 2, the obtained results show some improvements which indicate the benefit of transferring the learned filters across the recognition tasks. Figure 6b shows the superiority of performance for the shallower models across all ranks.

**Fine-Tuning Pretrained Models:** The obtained results suggest that fine-tuning pretrained networks on the target task is a preferable option. As expected, the evaluation metrics are higher under this learning strategy. Despite the improvement in all metrics for all models, deeper models maximize their benefit and achieve a better performance. The models achieve R1 rates of above 71% and the best performance is obtained by the VGG-19 which indicates a significant improvement of above 74% in the R1 rate. Therefore, adjusting the convolutional kernels in the feature extraction block along with the classification part leads to more discriminative features and superior performance. To better visualize the performance variations across the different models, the CMC curves are plotted in Figure 6c. We notice an improvement in all performance curves with a higher recognition rate for the VGG-19 in the first five ranks followed by VGG-16 and VGG-13 in the higher ranks.

**Ensembles of Models:** We start our ensembles by combining the VGG-13 and VGG-16 as both have an identical performance of nearly 74% for R1. The result is an improved R1 recognition rate above 2%. In addition, adding the VGG-19 model to the ensemble, leads to a further improvement in the R1 and R5 recognition rates of 2% and 1%, respectively. Furthermore, adding the VGG-11 to the triplet of models enhances the performance and yields the best R1 recognition rate, which exceeds 79%. The obtained results from these ensembles indicate the advantage of having multiple image representations learned by different models to address the unconstrained ear recognition problem and improve the overall performance.

### 5.4. Visualization of the Obtained Models

Although deep learning techniques achieve state-of-the-art results, understanding and interpreting the obtained results and the reason behind network decisions are of paramount importance. In this section, we seek to better interpret what deep models have learned after training using the Grad-CAM visualization technique. We illustrate the representation learned by highlighting the regions of interest (areas where the models focus) when making a prediction decision. Even though similar conclusions are obtained for the other models, for ease of exploration, we choose the VGG-16 models obtained after training on both the AMI and AMIC datasets and visualize all the correctly classified subjects as well as the misclassified ones from both datasets, as shown in Table 3 and Table 4.

The Grad-CAM approach finds and highlights spatial image regions that strongly contribute to making a specific decision, by providing a heatmap. Thus, in Table 3, we present both the original image and the heatmap extracted by Grad-CAM, with black pixels indicating the least relevant regions and white pixels indicating the most relevant regions. In addition, the original image is combined with the heatmap to form one comprehensive image using the Hue, Saturation, Value (HSV) colormap, where red-yellow denotes regions of low relevance and purple-red denotes regions of high relevance. It appears to be a convenient phenomenon that models are able to make a correct prediction when they focus on the geometric structure of the ear, although they are free to utilize some other features as well.

In order to get an insight into the process of wrong ear identification, misclassified samples and their Grad-CAM visualizations are also added to Table 3. The network predominantly focuses on textures at the ear boundary when making wrong decisions and seems to overstate the importance of certain ear parts, the haircut, or skin texture. Nevertheless, this kind of auxiliary information is successfully utilized for other samples, as illustrated in Table 4. These manually chosen images unravel how the networks actually include features that are not part of the ear for their correct predictions.

### 5.5. Why Is the Recognition Rate Lower on the AMIC Dataset?

Our results in Table 2 clearly show that the shift from the original AMI dataset to its cropped counterpart is accompanied by a noticeable drop in performance. In this section, we try to interpret this phenomenon with the help of the Grad-CAM visualization technique. In the previous section, we found that models successfully consider other parts of the profile image, such as hair and neck, in making their predictions. A plausible reason for the performance drop is the cropping of the profile images, which may result in losing certain valuable information or some specific region that helps the models obtain a correct prediction. Table 3 presents visualizations of some correctly identified samples that were drawn from the AMIC image set, and for the purpose of direct comparison we also present some misclassified images. One can observe that models have no other choice than to focus on the shape and texture of the ear.

Nevertheless, wrong decisions seem to be somewhat correlated to a stronger focus on regions close to the image boundary. It is possible that filter outputs located at the border of a spacial feature map suffer more from incomplete convolution (padding) than filter outputs corresponding to the center of a feature map. As a result, the receptive field needs more layers to actually cover some relevant part of the ear. Thus, relying on far-from-center regions might be somewhat less robust leading to a higher chance of misclassification. However, conducting the experiments with no padding actually did not further increase the performance metrics.

### 5.6. Computational Complexity

As a result of the fact that error back-propagation requires a massive amount of numerical operations, model optimizations need to be taken into considerations as the small memory footprint and high speed of the obtained models are of paramount importance, especially if one intends to deploy the algorithm on devices with limited computational resources. As an additional step to reduce the computation time, we reduce the input image size to 133×190= 25,270 for both AMI and AMIC and 170×220 for the WPUT, which is much smaller than the 224×224 pixels required for training on ImageNet dataset. Making the images even smaller leads to a decrease in the performance metrics. As a result, we found these image sizes to be the optimal ones in combination with our data augmentation methodology and the VGG architecture.

Table 5 shows important factors of the different VGG network-based models including model size, number of trainable parameters, size of the feature maps of the fully connected layers, training time, and batch size. The training time is measured in seconds per batch. We notice from Table 5 that the number of trainable parameters is smaller compared to the original VGG architectures for two main reasons. First, the VGG network was proposed to tackle the ImageNet classification task which has 1000 classes; however, our datasets have only 100 and 474 classes and consequently less parameters in the fully connected layers. Second, we perform adjustments to the original VGG architectures to suit our recognition task and further reduce the number of trainable parameters.

## 6. Conclusions and Future Work

The aim of this work was to address the ear identification problem under constrained and unconstrained conditions using deep learning models of increasing depth and ensembles of the best models as well as to improve the recognition performance. We considered four VGG-like network architectures and three ear image representation learning strategies including: training networks with randomly initialized weights, feature extraction using pretrained networks, and fine-tuning models which were pretrained on image classification tasks. The experimental results indicate the supremacy of the fine-tuned networks on ear images compared to the other learning methods. Furthermore, the recognition performance was further improved, independent of the dataset size, when constructing ensembles of the fine-tuned networks. Despite the improved performance on all datasets with the ensembles, the improvement under uncontrolled conditions was higher due to the wide variations in images, meaning that the different models could learn more distinguishing features. Therefore, it is of a crucial importance to utilize multiple representations for ear images acquired under uncontrolled settings. Experimentally, the proposed ensembles achieved the best recognition accuracy on all datasets with a large margin of at least 11% and 6% under constrained and unconstrained conditions, respectively.

In order to understand what the deep models have learned and attempting to interpret the obtained results and improve ear recognition understanding, we provide visualizations of the most discriminative regions in the ear image which lead to correct or false predictions. With the Grad-CAM visualization technique, we were able to see where the models focus when making their predictions. On the basis of the results from the visualization experiments, we found that focusing on the ear shape is frequently associated with correct predictions; while considering other parts of the profile image can result in misleading predictions. On the other hand, utilizing auxiliary parts of profile images could help in distinguishing certain high-level features like gender or haircut based on visible parts of the hair or neck. Therefore, we introduced a cropped ear image set (AMIC) based on the existing profile images from the AMI dataset. The main purpose of the introduced dataset was: (i) to make the networks process only the ear shape and structure so that they can learn only the relevant ear features; (ii) to build trust in the obtained results based on exclusively seeing the ear which is the main aim of the ear recognition study; and (iii) to evaluate the performance of newly proposed ear recognition systems in the future.

Even though the obtained results are promising and significantly improve the recognition performance on the considered datasets, there is still room for improvement, such as overcoming the limitations of small-scale ear datasets, and efficiently learning features that exhibit robustness to various image transformations. In future work, we plan to extend our experiments to explore various deep CNNs architectures and suitable learning strategies, whilst focusing more on the problem of unconstrained ear recognition. On the other hand, we will extend our study as regards different CNN feature visualization techniques to understand what the network or certain layers learn in order to find the most relevant regions of the human ear. A very interesting point for future study is the exploration of ways to control the attention of CNNs, allowing the networks focus only on certain discriminative regions in the input image in order to improve their learning ability.

## Figures and Tables

**Table 1:** Details of the visual geometry group (VGG)-based networks configurations with respect to number and type of layers (depth), filter size, number of filters, and the output size after each operation. The input image size and output size shown in the table are for the mathematical analysis of images (AMI) and AMI cropped (AMIC) datasets. For the West Pomeranian University of Technology (WPUT) dataset, the models use an input image size of 170×220 to preserve the aspect ratio and the output after each operation is obtained similar to AMI and AMIC.

Block	Model				Filter Size	Output Size
	VGG-11	VGG-13	VGG-16	VGG-19		
Input	Input Image (133×190 RGB)				-	-
Block 1	Convolution	Convolution	Convolution	Convolution	3×3 (64)	133×190
		Convolution	Convolution	Convolution	3×3 (64)	133×190
	Max-Pooling				-	66×95
Block 2	Convolution	Convolution	Convolution	Convolution	3×3 (128)	66×95
		Convolution	Convolution	Convolution	3×3 (128)	66×95
	Max-Pooling				-	33×47
Block 3	Convolution	Convolution	Convolution	Convolution	3×3 (256)	33×47
	Convolution	Convolution	Convolution	Convolution	3×3 (256)	33×47
			Convolution	Convolution	3×3 (256)	33×47
				Convolution	3×3 (256)	33×47
	Max-Pooling				-	16×23
Block 4	Convolution	Convolution	Convolution	Convolution	3×3 (512)	16×23
	Convolution	Convolution	Convolution	Convolution	3×3 (512)	16×23
			Convolution	Convolution	3×3 (512)	16×23
				Convolution	3×3 (512)	16×23
	Max-Pooling				-	8×11
Block 5	Convolution	Convolution	Convolution	Convolution	3×3 (512)	8×11
	Convolution	Convolution	Convolution	Convolution	3×3 (512)	8×11
			Convolution	Convolution	3×3 (512)	8×11
				Convolution	3×3 (512)	8×11
	Adaptive Average Pooling				-	5×5
Fully Connected	Fully Connected, 2048 Neurons					
	Dropout Chance 50%					
	Fully Connected, 2048 Neurons					
	Dropout Chance 50%					
	Fully Connected, 100 Neurons					
	Log-Soft-Max					

**Table 2:** A comparison of rank-one (R1), rank-five (R5), and area under cumulative match characteristic (AUC) for the different VGG-based models on the AMI, its cropped version AMIC, and WPUT ear datasets. The results are given in percentages and the top three metrics for each learning method on each dataset are highlighted in bold. The reported results from our ensembles on the AMI and WPUT datasets indicate significant improvements of recognition rates over recent studies in the literature.

Methods	Model	AMI			AMIC			WPUT		
		R1	R5	AUC	R1	R5	AUC	R1	R5	AUC
Scratch Training	VGG-11	83.21	97.14	98.57	69.28	87.14	96.61	47.83	71.68	96.40
	VGG-13	84.28	96.07	98.51	70.35	87.86	96.70	46.56	69.26	96.24
	VGG-16	86.07	96.79	98.54	70.35	84.29	96.34	46.17	68.75	96.20
	VGG-19	84.28	95.00	98.45	68.92	83.21	95.65	44.13	66.07	95.96
Feature Extraction	VGG-11	76.07	93.92	98.24	80.35	93.21	97.96	59.44	81.12	98.19
	VGG-13	81.78	97.50	98.61	71.78	89.64	97.01	51.53	76.15	97.56
	VGG-16	83.21	93.92	98.22	74.64	88.21	96.46	50.13	73.72	97.54
	VGG-19	68.92	91.42	97.53	62.85	81.79	95.76	44.26	69.52	97.10
Fine-Tuning	VGG-11	94.29	98.93	98.83	89.64	95.36	98.28	71.05	86.61	98.56
	VGG-13	94.64	98.57	98.86	90.36	96.79	98.67	73.98	88.78	98.71
	VGG-16	96.07	99.29	98.87	89.29	97.50	98.42	73.98	89.16	98.70
	VGG-19	96.78	99.29	98.92	92.14	97.86	98.60	74.36	88.65	98.69
Our Ensembles	VGG-13-16	97.14	99.64	98.91	92.85	96.42	98.57	76.40	90.69	98.87
	VGG-13-16-19	97.50	99.64	98.41	92.85	97.85	98.67	78.19	91.07	98.89
	VGG-11-13-16-19	97.14	99.64	98.91	93.21	96.78	98.63	79.08	90.43	98.92
Previous Work	Chowdhury et al. [69]	67.26	-	-	-	-	-	65.15	-	-
		63.53	-	-	-	-	-	67.00	-	-
	Hassaballah et al. [9]	72.86	-	-	-	-	-	38.76	-	-
		73.71	-	-	-	-	-	37.13	-	-
	Alshazly et al. [10]	70.20	-	-	-	-	-	-	-	-
	Raghavendra et al. [70]	86.36	-	-	-	-	-	-	-	-
	Sultana et al. [71]	-	-	-	-	-	-	73.00	86.00	-

**Table 3:** Qualitative examples of gradient-weighted class activation map (Grad-CAM) visualization results for four correctly classified and four misclassified ear images from each ear dataset. The results were generated using the VGG-16 model trained with random weight initialization. For a correct classification, the model considers some discriminative regions and more specifically the geometric structure of the ear shape, while misclassification seems to be the result of utilizing misleading regions such as the auxiliary parts of the hair and neck.

Dataset	Correctly Classified			Misclassified		
	Original	Grad-CAM	Heatmap	Original	Grad-CAM	Heatmap
**AMI**	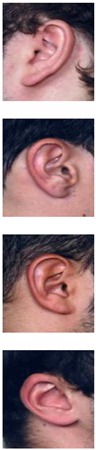	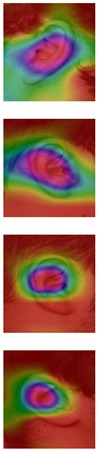	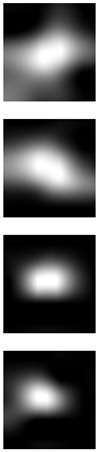	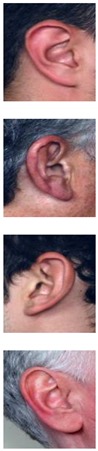	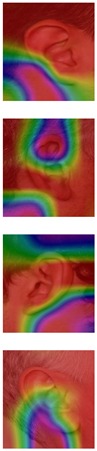	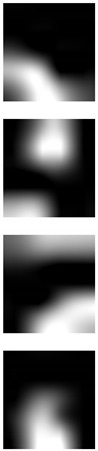
**AMIC**	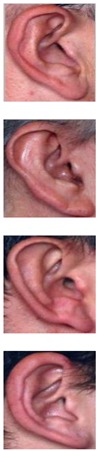	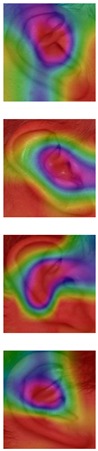	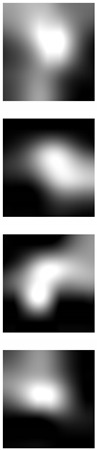	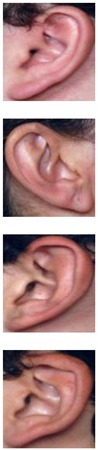	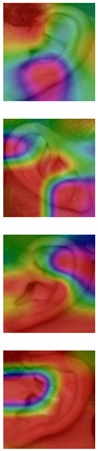	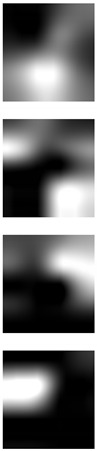

**Table 4:** Grad-CAM visualization of correctly classified subjects from the AMI dataset. The model looks at various places around the ear structure, exploiting these auxiliary parts to make predictions. This is justifying the drop in recognition rate on the AMIC dataset where these extra parts are removed from the profile images.

Dataset	Original	Grad-CAM	Heatmap	Original	Grad-CAM	Heatmap
**AMI**	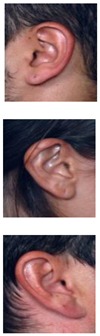	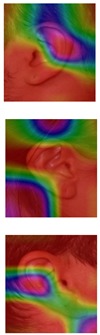	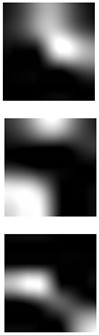	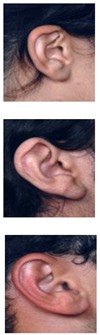	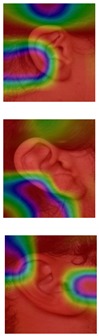	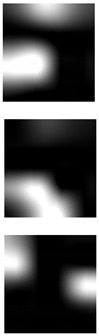

**Table 5:** A comparison of time and memory consumption by the various VGG models along with other distinguishing characteristics. The analysis is conducted using the AMI dataset.

Model	Model Size (MB)	Trainable Parameters	Feature Size	Training Time (Seconds Per Batch)	Batch Size
VGG-11	151.99	39,843,684	2048	3.6	25
VGG-13	152.70	40,028,580	2048	3.7	25
VGG-16	172.96	45,340,836	2048	3.7	25
VGG-19	193.23	50,653,092	2048	3.8	25

## Abbreviations

The following abbreviations are used in this manuscript:

CNN	Convolutional Neural Network
USTB	University of Science and Technology Beijing
AWE	Annotated Web Ear
ReLU	Rectified Linear Unit
HSV	(Hue, Saturation, Value)
AMI	Mathematical Analysis of Images
AMIC	AMI Cropped
VGG	Visual Geometry Group
CAM	Class Activation Map
Grad-CAM	Gradient-weighted Class Activation Map
CMC	Cumulative Match Characteristic
R1	Rank 1
R5	Rank 5
AUC	Area Under CMC
WPUT	West Pomeranian University of Technology

## References

[1] Jain A.K., Ross A., Prabhakar S. (2004). An introduction to biometric recognition. IEEE Trans. Circuits Syst. Video Technol..

[2] Iannarelli A.V. (1989). Ear Identification.

[3] Pflug A., Busch C. (2012). Ear biometrics: A survey of detection, feature extraction and recognition methods. IET Biom..

[4] Abaza A., Ross A., Hebert C., Harrison M.A.F., Nixon M.S. (2013). A survey on ear biometrics. ACM Comput. Surv..

[5] Emeršič Ž., Štruc V., Peer P. (2017). Ear recognition: More than a survey. Neurocomputing.

[6] Galdámez P.L., Raveane W., Arrieta A.G. (2017). A brief review of the ear recognition process using deep neural networks. J. Appl. Log..

[7] Benzaoui A., Hadid A., Boukrouche A. (2014). Ear biometric recognition using local texture descriptors. J. Electron. Imaging.

[8] Omara I., Wu X., Zhang H., Du Y., Zuo W. (2018). Learning pairwise SVM on hierarchical deep features for ear recognition. IET Biom..

[9] Hassaballah M., Alshazly H.A., Ali A.A. (2019). Ear recognition using local binary patterns: A comparative experimental study. Expert Syst. Appl..

[10] Alshazly H.A., Hassaballah M., Ahmed M., Ali A.A. Ear Biometric Recognition Using Gradient-Based Feature Descriptors. Proceedings of the International Conference on Advanced Intelligent Systems and Informatics.

[11] Pflug A., Paul P.N., Busch C. A comparative study on texture and surface descriptors for ear biometrics. Proceedings of the International Carnahan Conference on Security Technology.

[12] Emeršič Z., Meden B., Peer P., Štruc V. Covariate analysis of descriptor-based ear recognition techniques. Proceedings of the International Conference and Workshop on Bioinspired Intelligence (IWOBI).

[13] Zhou J., Cadavid S., Abdel-Mottaleb M. Exploiting color SIFT features for 2D ear recognition. Proceedings of the International Conference on Image Processing.

[14] Nanni L., Lumini A. (2009). Fusion of color spaces for ear authentication. Pattern Recognit..

[15] Bustard J.D., Nixon M.S. (2010). Toward unconstrained ear recognition from two-dimensional images. IEEE Trans. Syst. Man Cybern. Part A Syst. Hum..

[16] LeCun Y., Bengio Y., Hinton G. (2015). Deep learning. Nature.

[17] Krizhevsky A., Sutskever I., Hinton G.E. ImageNet classification with deep convolutional neural networks. Proceedings of the 25th International Conference on Neural Information Processing Systems.

[18] Simonyan K., Zisserman A. (2014). Very deep convolutional networks for large-scale image recognition. arXiv.

[19] Szegedy C., Vanhoucke V., Ioffe S., Shlens J., Wojna Z. Rethinking the inception architecture for computer vision. Proceedings of the IEEE Conference on Computer Vision and Pattern Recognition.

[20] Ren S., He K., Girshick R., Sun J. Faster R-CNN: Towards real-time object detection with region proposal networks. Proceedings of the Advances in Neural Information Processing Systems.

[21] Girshick R., Donahue J., Darrell T., Malik J. Rich feature hierarchies for accurate object detection and semantic segmentation. Proceedings of the IEEE Conference on Computer Vision and Pattern Recognition.

[22] Redmon J., Divvala S., Girshick R., Farhadi A. You Only Look Once: Unified, real-time object detection. Proceedings of the IEEE Conference on Computer Vision and Pattern Recognition.

[23] Schroff F., Kalenichenko D., Philbin J. FaceNet: A unified embedding for face recognition and clustering. Proceedings of the IEEE Conference on Computer Vision and Pattern Recognition.

[24] Parkhi O.M., Vedaldi A., Zisserman A. Deep Face Recognition. Proceedings of the British Machine Vision Conference.

[25] Wen Y., Zhang K., Li Z., Qiao Y. A discriminative feature learning approach for deep face recognition. Proceedings of the European Conference on Computer Vision.

[26] Taigman Y., Yang M., Ranzato M., Wolf L. Deepface: Closing the gap to human-level performance in face verification. Proceedings of the IEEE Conference on Computer Vision and Pattern Recognition.

[27] Deng J., Dong W., Socher R., Li L.J., Li K., Fei-Fei L. ImageNet: A Large-scale Hierarchical Image Database. Proceedings of the IEEE Conference on Computer Vision and Pattern Recognition.

[28] Emeršič Ž., Štepec D., Štruc V., Peer P. Training convolutional neural networks with limited training data for ear recognition in the wild. Proceedings of the International Conference on Automatic Face and Gesture Recognition.

[29] Iandola F.N., Han S., Moskewicz M.W., Ashraf K., Dally W.J., Keutzer K. (2016). SqueezeNet: AlexNet-level accuracy with 50x fewer parameters and <0.5 MB model size. arXiv.

[30] Ying T., Shining W., Wanxiang L. Human ear recognition based on deep convolutional neural network. Proceedings of the 30th Chinese Control And Decision Conference (CCDC).

[31] Tian L., Mu Z. Ear recognition based on deep convolutional network. Proceedings of the International Congress on Image and Signal Processing, BioMedical Engineering and Informatics (CISP-BMEI).

[32] Yuan L., Liu W., Li Y. (2016). Non-negative dictionary based sparse representation classification for ear recognition with occlusion. Neurocomputing.

[33] Pan S.J., Yang Q. (2010). A survey on transfer learning. IEEE Trans. Knowl. Data Eng..

[34] Sharif Razavian A., Azizpour H., Sullivan J., Carlsson S. CNN Features off-the-shelf: An Astounding Baseline for Recognition. Proceedings of the Computer Vision and Pattern Recognition Workshops.

[35] Yosinski J., Clune J., Bengio Y., Lipson H. (3320–3328). How transferable are features in deep neural networks? In Proceedings of the 27th International Conference on Neural Information Processing Systems, Montreal, QC, Canada, 8–13 December, 2014; pp.

[36] Almisreb A.A., Jamil N., Din N.M. Utilizing AlexNet Deep Transfer Learning for Ear Recognition. Proceedings of the 4th International Conference on Information Retrieval and Knowledge Management (CAMP).

[37] Zhang Y., Mu Z., Yuan L., Yu C. (2018). Ear verification under uncontrolled conditions with convolutional neural networks. IET Biom..

[38] Eyiokur F.I., Yaman D., Ekenel H.K. (2017). Domain adaptation for ear recognition using deep convolutional neural networks. IET Biom..

[39] Zhang Y., Mu Z., Yuan L., Yu C., Liu Q. USTB-Helloear: A large database of ear images photographed under uncontrolled conditions. Proceedings of the International Conference on Image and Graphics.

[40] Dodge S., Mounsef J., Karam L. (2018). Unconstrained ear recognition using deep neural networks. IET Biom..

[41] Hansley E.E., Segundo M.P., Sarkar S. (2018). Employing fusion of learned and handcrafted features for unconstrained ear recognition. IET Biom..

[42] Emeršič Ž., Meden B., Peer P., Štruc V. (2018). Evaluation and analysis of ear recognition models: performance, complexity and resource requirements. Neural Comput. Appl..

[43] Emeršič Ž., Štepec D., Štruc V., Peer P., George A., Ahmad A., Omar E., Boult T.E., Safdaii R., Zhou Y. The Unconstrained Ear Recognition Challenge. Proceedings of the International Joint Conference on Biometrics.

[44] Emeršič Ž., Harish B., Gutfeter W., Khiarak J.N., Pacut A., Hansley E., Segundo M.P., Sarkar S., Park H., Nam G.P. The Unconstrained Ear Recognition Challenge 2019. Proceedings of the International Conference on Biometrics.

[45] Kacar U., Kirci M. (2018). ScoreNet: Deep cascade score level fusion for unconstrained ear recognition. IET Biom..

[46] Yosinski J., Clune J., Nguyen A., Fuchs T., Lipson H. (2015). Understanding neural networks through deep visualization. arXiv.

[47] Zeiler M.D., Fergus R. Visualizing and understanding convolutional networks. Proceedings of the European Conference on Computer Vision.

[48] Springenberg J.T., Dosovitskiy A., Brox T., Riedmiller M. (2014). Striving for Simplicity: The All Convolutional Net. arXiv.

[49] Zhou B., Khosla A., Lapedriza A., Oliva A., Torralba A. Learning deep features for discriminative localization. Proceedings of the IEEE Conference on Computer Vision and Pattern Recognition.

[50] Simonyan K., Vedaldi A., Zisserman A. (2013). Deep inside convolutional networks: Visualising image classification models and saliency maps. arXiv.

[51] Selvaraju R.R., Cogswell M., Das A., Vedantam R., Parikh D., Batra D. Grad-CAM: Visual Explanations from Deep Networks via Gradient-based Localization. Proceedings of the International Conference on Computer Vision (ICCV).

[52] Russakovsky O., Deng J., Su H., Krause J., Satheesh S., Ma S., Huang Z., Karpathy A., Khosla A., Bernstein M. (2015). ImageNet large scale visual recognition challenge. Int. J. Comput. Vis..

[53] LeCun Y., Bottou L., Bengio Y., Haffner P. (1998). Gradient-based learning applied to document recognition. Proc. IEEE.

[54] Nair V., Hinton G.E. Rectified linear units improve restricted boltzmann machines. Proceedings of the 27th International Conference on International Conference on Machine Learning (ICML).

[55] Srivastava N., Hinton G., Krizhevsky A., Sutskever I., Salakhutdinov R. (2014). Dropout: A simple way to prevent neural networks from overfitting. J. Mach. Learn. Res..

[56] Garcia-Gasulla D., Vilalta A., Parés F., Ayguadé E., Labarta J., Cortés U., Suzumura T. An out-of-the-box full-network embedding for convolutional neural networks. Proceedings of the International Conference on Big Knowledge (ICBK).

[57] Kornblith S., Shlens J., Le Q.V. (2018). Do better ImageNet models transfer better?. arXiv.

[58] Ge W., Yu Y. Borrowing treasures from the wealthy: Deep transfer learning through selective joint fine-tuning. Proceedings of the IEEE Conference on Computer Vision and Pattern Recognition.

[59] Donahue J., Jia Y., Vinyals O., Hoffman J., Zhang N., Tzeng E., Darrell T. Decaf: A deep convolutional activation feature for generic visual recognition. Proceedings of the 31st International Conference on Machine Learning.

[60] Hertel L., Barth E., Käster T., Martinetz T. Deep convolutional neural networks as generic feature extractors. Proceedings of the International Joint Conference on Neural Networks.

[61] Guo J., Gould S. (2015). Deep CNN ensemble with data augmentation for object detection. arXiv.

[62] Ahmad K., Mekhalfi M.L., Conci N., Melgani F., Natale F.D. (2018). Ensemble of deep models for event recognition. ACM Trans. Multimed. Comput. Commun. Appl..

[63] Rumelhart D.E., Hinton G.E., Williams R.J. (1988). Learning representations by back-propagating errors. Cogn. Model..

[64] Gonzalez E. AMI Ear Database. http://www.ctim.es/research_works/ami_ear_database.

[65] Frejlichowski D., Tyszkiewicz N. The West Pomeranian University of Technology Ear Database—A Tool for Testing Biometric Algorithms. Proceedings of the International Conference on Image Analysis and Recognition.

[66] Cireşan D., Meier U., Schmidhuber J. (2012). Multi-column deep neural networks for image classification. arXiv.

[67] Howard A.G. (2013). Some improvements on deep convolutional neural network based image classification. arXiv.

[68] Dellana R., Roy K. Data augmentation in CNN-based periocular authentication. Proceedings of the 6th International Conference on Information Communication and Management (ICICM 2016).

[69] Chowdhury D.P., Bakshi S., Guo G., Sa P.K. (2018). On Applicability of Tunable Filter Bank Based Feature for Ear Biometrics: A Study from Constrained to Unconstrained. J. Med. Syst..

[70] Raghavendra R., Raja K.B., Busch C. Ear recognition after ear lobe surgery: A preliminary study. Proceedings of the IEEE International Conference on Identity, Security and Behavior Analysis (ISBA).

[71] Sultana M., Polash Paul P., Gavrilova M. (2015). Occlusion detection and index-based ear recognition. J. WSCG.

[72] Blumer A., Ehrenfeucht A., Haussler D., Warmuth M.K. (1987). Occam’s razor. Inf. Process. Lett..

